# 

**Published:** 1893-03

**Authors:** 


VOL. XIV.] *PUBLISHED MONTHLY AT THREE DOLLARS A YEAR. SINGLE COPIES, THIRTY CENTS.* [No. 3.

Entered at the Post Office, February 3rd, 1891, at New York, as Second-class Matter.
Copyright, 1893, by W. A. Conklin and R. S. Huidekoper.
Printed for the Editors by E. SCOTT, 134 West 23d Street, New York.

### THE JOURNAL OF COMPARATIVE MEDICINE AND VETERINARY ARCHIVES.

EDITED BY

W. A. CONKLIN, Ph. D., D. V. S.

RUSH SHIPPEN HUIDEKOPER, M. D., Veterinarian, (Alfort),

*NEW YORK.*

MARCH, 1893.

All communications and payments should be addressed to

**MANAGING EDITOR JOURNAL COMP. MEDICINE AND VET. ARCH.**

**Station G, New York City.**

**Copies may be had in London of BALLIERE, TINDALL & COX.**

